# Mechanical Properties Degradation of Fiberglass Tubes during Biaxial Proportional Cyclic Loading

**DOI:** 10.3390/polym15092017

**Published:** 2023-04-24

**Authors:** Valeriy Wildemann, Oleg Staroverov, Elena Strungar, Artur Mugatarov, Artur Kuchukov

**Affiliations:** Center of Experimental Mechanics, Perm National Research Polytechnic University, 614990 Perm, Russia; cem_staroverov@mail.ru (O.S.); cem.spaskova@mail.ru (E.S.); cem_mugatarov@mail.ru (A.M.); artur.kuchukov.59@mail.ru (A.K.)

**Keywords:** composite, tubular sample, multiaxial fatigue, damage accumulation, residual mechanical properties

## Abstract

Composite structures during an operation are subjected to various types of external loading (impact, vibration, cyclic, etc.), which may lead to a decrease in mechanical properties. Previously, many experimental investigations of the mechanical behavior of composites under uniaxial cyclic loading were carried out. Acquisition of new data on the reduction of composite materials’ mechanical characteristics under conditions of multiaxial cyclic loading, as well as verification of existing models for calculation of the residual properties, are relevant. Therefore, this work is devoted to the experimental investigation of the mechanical behavior of fiberglass tubes under proportional cyclic loading. Static and fatigue tests were carried out under tension with torsion conditions. Inhomogeneous strain fields were obtained using a non-contact optical video system VIC-3D. The structural damage accumulation processes were analyzed by an AMSY-6 acoustic emission signals recording system. Surface defects were determined using a DinoLite microscope. Residual dynamic elastic modules were calculated during fatigue tests, and fatigue sensitivity curves were built. Data was approximated using various models, and their high descriptive capability was revealed. Damage accumulation stages were determined. The dependence of the models’ parameters on a stress state were observed. It was concluded that multiaxial cyclic loading leads to a significant decrease in mechanical properties, which should be taken into account in composite structure design.

## 1. Introduction

Composite materials allow the problem of maintaining a high level of physical and mechanical characteristics while reducing the structure weight to be solved in industries such as space, aviation, automotive, construction, etc. Trends demonstrate that the development of technology for the production of the composite materials based on a polymer matrix and the growth of their usage in designs [1,2,3,4,5,6].

Every unforeseen impact, vibration, or other load, even of low intensity and duration, can lead to a significant reduction in the exploitation time [7,8,9,10,11,12]. In this regard, it is relevant to investigate patterns of the degradation of composites’ mechanical properties under complex and combined loads. The study of the cyclic exposure influence on the residual mechanical characteristics of composites, called fatigue sensitivity, is one of the most significant.

A large number of works on this topic are devoted to the experimental investigation of the degradation of the polymer composites’ mechanical properties (elasticity modulus and tensile strength) during uniaxial cyclic loading [13,14,15,16,17,18,19]. On the other hand, the overwhelming majority of composite structures are in a complex stress–strain state, so it is necessary to study fatigue sensitivity of composites under multiaxial cyclic loading. In this case, many loading parameters should be taken into account, including the ratio of the amplitudes of the stress tensor components [20,21,22], the phase shift angle between the loading modes [23], the frequencies ratio [24,25], the mean stress, and the stress amplitude in the cycle [26,27,28,29].

Recent testing systems allow the implementation of a complex stress–strain state by loading cruciform specimens in two orthogonal axes [30,31,32], the tension of tubular specimens under the internal pressure [33,34], the torsion of tubular specimens with bending [35,36,37,38], and tension–compression with torsion [39,40,41]. Experimental studies of the degradation of the composites’ mechanical properties under multiaxial cyclic loading have been presented previously [42,43,44]. The residual characteristics assessment is carried out by calculation of the elastic modulus during the fatigue test.

The relationship between the mechanical characteristics and preliminary cyclic exposure can be represented as the fatigue sensitivity curve. As demonstrated previously [45], these curves have three characteristic stages: initiation, where intense damage accumulation processes occur, leading to rapid degradation of properties; stabilization, where damage accumulation is slow, and the stage is almost linear; and aggravation, where intense damage accumulation processes lead to a complete loss of bearing capacity. Previously [45,46], it was proposed to determine the boundaries of these stages by the points where the damage value function derivative reaches a certain value (characteristic to the material).

The experimentally obtained fatigue sensitivity curves are necessary to build and verify residual mechanical properties prediction models. Some of them were proposed previously [47,48,49,50,51]. However, these models are often only suitable for a particular class of composite and require the definition of many parameters. Mao and Mahadevan [52] proposed the convenient approximation of the fatigue sensitivity curve as a sum of two power functions. The authors of this study previously proposed two models based on the usage of cumulative probability distribution functions [46]. The advantages of these models are simplicity and few parameters, which means a reduction in the required experimental tests.

Nevertheless, an understanding of the damage accumulation processes requires usage of additional diagnostic systems. At first, the non-contact optical video system and digital image correlation method (DIC) allow the observation of inhomogeneous strain fields on the sample surface. DIC technology showed an advantage in the analysis of the stress–strain state of composite objects [53,54], the formation and propagation of cracks [55], the detection of defects, and the quantitative assessment of displacements and deformations [56]. Compared to traditional strain gauges [57] and finite element simulations [58], DIC can obtain more accurate and complete information about displacement and strain fields across the entire surface, not limited by sample geometry [59]. Moreover, since the destruction of polymer composites is a complex process that occurs at different scale levels, the identification of damages is necessary to predict the failure mechanism. The acoustic emission method allows the estimation of the intensity of damage accumulation and establishment of a connection with the composites’ structural destruction mechanisms by analyzing the frequency spectrum of the signals [60,61,62,63,64,65]. For verification of the acoustic emission signal analysis, optical microscopy is also required.

In this study, we investigate the degradation of mechanical properties during biaxial static and cyclic loading. The main goals of this work were to obtain new experimental data on stiffness degradation of fiberglass thin-walled tubes (produced by oblique transverse longitudinal continuous winding) and to verify previously proposed fatigue sensitivity curve approximation models. The investigation includes usage of the non-contact video system, acoustic emission system, and optical microscope.

The paper is organized as follows: the material, equipment, methods, and approximation models are described in Section 2; Section 3.1 presents the quasistatic tests results; fatigue experiments results and approximations of fatigue sensitivity curves are discussed in Section 3.2; and Section 4 closes the paper with the main conclusions of the work.

## 2. Material and Methods

### 2.1. Material

Tubes based on EC glass fiber (1200 tex, ≈580–600 N breaking load value) and cold curing epoxy resin KER 828 were made by oblique transverse longitudinal continuous winding at an angle of 85° to the axis (this angle is often used in the production of pipes, which work under internal pressure). The winding tapes consisted of fibers lying along the tape and had few fibers, which were oriented across it. For testing, thin-walled fiberglass tubular samples were prepared. The sample length was 140 mm with the working part length *L* ≈ 60 mm, inner diameter *d* = 25.4 mm, and outer diameter *D* ≈ 30 mm.

### 2.2. Equipment

Experimental studies were carried out using the large-scale research facilities “Complex of testing and diagnostic equipment for studying properties of structural and functional materials under complex thermomechanical loading” at the Center of Experimental Mechanics of the Perm National Research Polytechnic University (PNRPU).

Quasistatic and cyclic tests were carried out using the Instron 8802 (±100 kN) universal servohydraulic testing system. The loading was recorded by a load cell up to 100 kN and 1000 N∙m. The loading measurement accuracy is 0.5%. The testing system included a FastTrack controller. The WaveMatrix V. 1.4 software allowed the implementation of biaxial loading modes.

**Figure 1:**
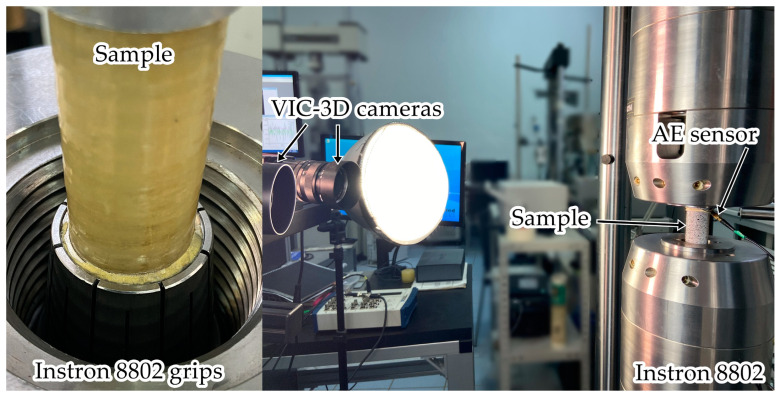
Sample in the testing machine grips and diagnostic systems.

Collet grips of the test system Instron 8802 were used to set samples (Figure 1, left side). Cylindrical steel plugs were inserted for the entire length of the gripping parts to prevent specimen crushing. When the sample was set, the pressure in the servo-hydraulic grip circuit gradually increased from 50 to 200 bar. To prevent the occurrence of overloads during fixation, the values of the axial load and torsional were kept at zero (“Specimen Protect” was on).

Displacements and strains of the specimen surface were recorded using the VIC-3D contactless optical video system (Correlated Solutions, Irmo, SC, USA) and the digital image correlation (DIC) method. A video recording was carried out using the camera Prosilica GE4900 50 mm with 16 MP resolution (Allied Vision, Stadtroda, Germany). The recording frequency was 1 frame per 3 s. The normalized sum of the squared difference (NSSD) was used as the correlation criterion for the mathematical assessment of the digital image correspondence. The strains were calculated using the Lagrange finite strain tensor. Transition from the Cartesian to cylindrical coordinate system, associated with specimen axis, was carried out using VIC-3D software (Vic-Snap Image Acquisition V. 9).

The acoustic emission signals were recorded using the AMSY-6 system (Vallen Systeme GmbH, Wolfratshausen, Germany). We employed a wideband sensor AE144A (Fujicera, Fujinomiya, Japan) with a frequency range of 100–500 kHz, a M31 (Fujicera, Fujinomiya, Japan) sensor with the frequency range of 300–800 kHz, and a preamplifier with a gain of 34 dB. The sensors were attached to the sample using a rubber fixture. The data sampling frequency was 10 MHz, and the threshold value for recording AE signals was 40 dB. The energy parameter and the frequency of the spectral maximum (characteristic of the fast Fourier transform) were considered as informative. The energy parameter of AE signals was calculated using a special software option in energy units (eu), 1 eu = 10^−14^ V^2^∙s. The AMSY-6 and the video system were synchronized with the test system controller using a 16-bit high-speed NI USB-6251 ADC unit. A photo of the diagnostic systems is shown in Figure 1 (right side).

After testing, we used a Dino-Lite microscope (AnMo Electronics Corporation, New Taipei City, Taiwan) with DinoCapture 2.0 software in order to determine the sample surface defects.

### 2.3. Methods

Eighteen fiberglass tubular samples were divided into two groups for quasi-static and cyclic tensile, torsion, and proportional tension–torsion tests with various ratios of normal and shear stress tensor components. To determine the nominal values of the fracture load and torque, 6 samples were tested. The speed of the movable grip was 2 mm/min in the tensile (strain rate ≈ 0.033 min^−1^), and 20 deg/min in the torsion test (strain rate ≈ 0.087 min^−1^). In the proportional loading tests, 2 samples were fractured for each loading mode. The Proportional 1 mode corresponds to the speed of movement grip 1 mm/min (strain rate ≈ 0.017 min^−1^) and 20 deg/min; for the Proportional 2 mode, it is 2 mm/min and 20 deg/min. On some samples, video recording of the displacement and strain fields was carried out using the VIC-3D system, as well as the recording of acoustic emission signals using the AMSY-6 system (Table 1).

As a result of quasi-static tests, the dependences of load, *P*, on grip displacement, *u*, and torque, *M*, on torsion angle, *φ*, were determined. Normal stress, *σ*, longitudinal strain, *ε*, shear stress, *τ*, and shear strain, *γ* (taking into account the length change during tension), were calculated as:

(1) $$\sigma =\frac{4P}{\pi \left(D^{2}-d^{2}\right)};\;\varepsilon =\frac{u}{L};\;\tau =\frac{16DM}{\pi \left(D^{4}-d^{4}\right)};\;\gamma =\frac{\varphi D}{2\left(L+u\right)}=\frac{\varphi D}{2L\left(1+\varepsilon \right)}$$

For each of the cyclic loading modes, 2 samples were tested to fatigue failure (*R* = 0.1, frequency *v* = 1 Hz). Acoustic emission signals were recorded on one sample in each loading mode (Table 1). The maximum stress value during the cycle was equal to a half of the average maximum normal and shear stress values from the quasi-static tests.

To assess the mechanical properties reduction by changing the dynamic elasticity modulus, the peak maximum and minimum values of the load, torque, displacement, and torsion angle were recorded for every 1, 10, and 100 cycles. The values of the secant dynamic Young’s modulus, *E*′, and the shear modulus, *G*′, were determined by the formulas:

(2) $$E^{\prime }=\frac{P_{\max}-P_{\min}}{u_{\max}-u_{\min}}\frac{4L}{\pi \left(D^{2}-d^{2}\right)};\;G^{\prime }=\frac{M_{\max}-M_{\min}}{\varphi_{\max}-\varphi_{\min}}\frac{32L}{\pi \left(D^{4}-d^{4}\right)}$$

### 2.4. Approximation Models

According to the calculated dynamic elasticity moduli for the first loading cycle, which can be designated as *E*′_0_ and *G*′_0_, and durability for current loading mode, *N*_0_, fatigue sensitivity coefficients, *K_E_* and *K_G_*, as well as the relative value of preliminary cyclic exposure, *n*, can be calculated for cycle number, *N*:

(3) $$K_{E}=\frac{E^{\prime }}{{E^{\prime }}_{0}};\;K_{G}=\frac{G^{\prime }}{{G^{\prime }}_{0}};\;n=\frac{N}{N_{0}}$$

The approximation of experimentally obtained dependences, *K_E_*(*n*) and *K_G_*(*n*), was carried out by three models based on cumulative distribution function usage (Weibull law and beta distribution) [46] and that proposed by Mao and Mahadevan [52]. The approximating formulas, respectively, had the form:

(4) $$K_{E}=1-\lambda {\left(-\ln\left(1-n\right)\right)}^{\frac{1}{\kappa }};\;K_{E}=1-\frac{B_{n}\left(\alpha ,\beta \right)}{B\left(\alpha ,\beta \right)};\;K_{E}=1-qn^{m_{1}}-\left(1-q\right)n^{m_{2}}$$

The damage accumulation rate can be defined as the damage value function derivative *ω*′*_E_* (or *ω*′*_G_*):

(5) $${\omega^{\prime }}_{E}=\frac{\lambda }{\kappa }{\left(-\ln\left(1-n\right)\right)}^{\frac{1}{\kappa }-1}\frac{1}{1-n};\;{\omega^{\prime }}_{E}=\frac{n^{\alpha -1}{\left(1-n\right)}^{\beta -1}}{B\left(\alpha ,\beta \right)};\;{\omega^{\prime }}_{E}=qm_{1}n^{m_{1}-1}+\left(1-q\right)m_{2}n^{m_{2}-1}$$

The model parameters were obtained numerically.

## 3. Results and Discussion

### 3.1. Static Loading

#### 3.1.1. Loading Curves

**Figure 2:**
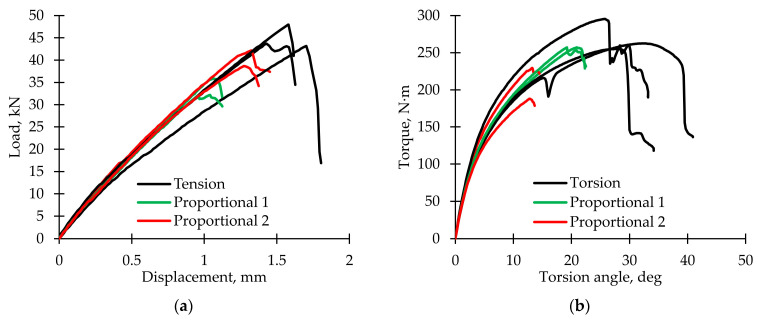
Loading curves. (**a**) Dependence of load on displacement. (**b**) Dependence of torque on torsion angle.

Loading curves (Figure 2) were built using the data from the test machine. It was noted that under tension, the diagram was almost linear to the maximum load value, and after the peak, a sharp drop was observed. This behavior corresponds to an elastic–brittle fracture. During torsion, the diagram was non-linear; the presence of a stage similar to plastic was noted. In addition, after reaching the peak, in some cases, a gradual torque decrease was observed, which means a postcritical stage realization [66]. Similar non-linear behavior of polymer composites under off-axis loading was considered previously [67,68,69]. For proportional loading modes, both the maximum load and the maximum torque value decrease. A two-fold slower grip movement in the Proportional 1 loading mode (in comparison with the Proportional 2 loading mode) led to a change in the ratio between normal and shear stresses; therefore, a lower load value and higher torque value were observed. Material sensitivity to the complex stress–strain state can be concluded.

#### 3.1.2. Failure Criterion

**Figure 3:**
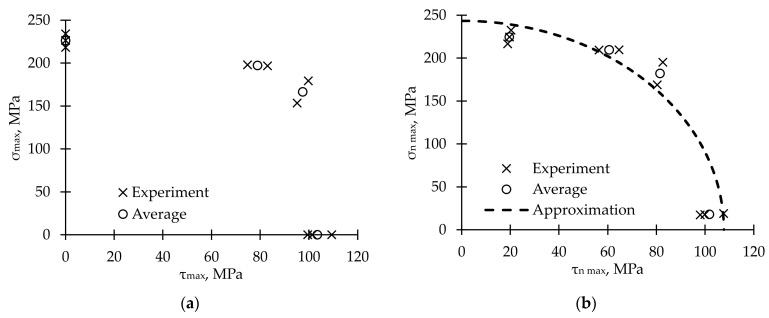
Maximum stress values. (**a**) In the global cylindrical coordinate system. (**b**) In a coordinate system associated with the winding angle.

Maximum values of normal stress, *σ*_max_, and shear stress, *τ*_max_, were calculated in the coordinate system, which was associated with sample axis, from the maximum values of the load and torque using Formula (1) (Figure 3a). However, since pipes were obtained by continuous winding, and their characteristic types of destruction is matrix cracking across the tape reinforcement and violations of adhesion between the fibers and tapes, it is rational to recalculate the stresses into a coordinate system associated with the direction of winding [70]. The formulas (for winding angle χ = 85°) are:

(6) $$\sigma_{n\;\max}=\sigma_{\max}\sin^{2}\chi +\tau_{\max}\sin2\chi ;\;\tau_{n\;\max}=\left|\frac{1}{2}\sigma_{\max}\sin2\chi +\tau_{\max}\cos2\chi \right|$$

If *τ_n_*_max_ = 0, *σ_n_*_max_ at the failure moment will be equal to the strength value denoted as *σ*_0*n* max_. Likewise, if *σ_n_*_max_ = 0, *τ_n_*_max_ will be equal to *τ*_0*n* max_ strength value. The failure criterion (similar to the 2-dimensional Hashin failure criterion in matrix tension mode [71]) was proposed as:

(7) $${\left(\frac{\sigma_{n\;\max}}{\sigma_{0n\;\max}}\right)}^{2}+{\left(\frac{\tau_{n\;\max}}{\tau_{0n\;\max}}\right)}^{2}=1$$

The criterion parameters were obtained numerically by experimental data approximation and were: *σ*_0*n* max_ = 243.4 MPa and *τ*_0*n* max_ = 107.7 MPa. A determination coefficient, *R*^2^, was 0.808, which means a good descriptive capability of this model. The experimental points and line that corresponded to criteria (7) are shown in Figure 3b. The proposed criteria can be used to define the failure of composite pipes obtained by winding.

#### 3.1.3. Inhomogeneous Displacement and Strain Field Analysis

**Figure 4:**
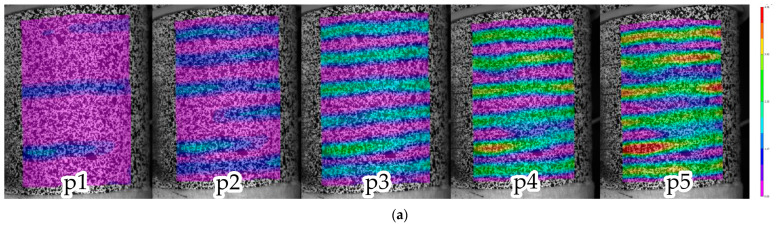

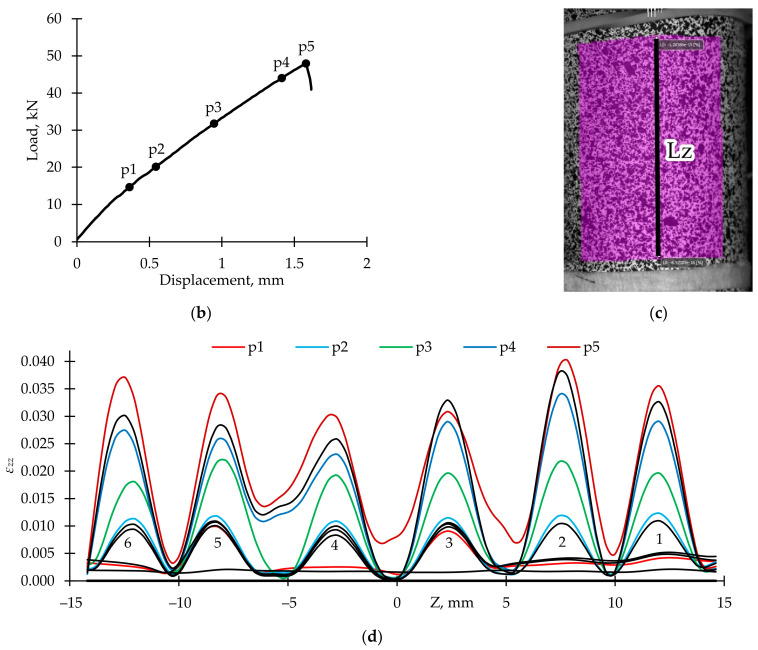
Inhomogeneous fields of longitudinal strain, *ε_zz_*, analysis. (**a**) Fields for load values p1–p5. (**b**) Loading diagram. (**c**) The line to build the strain diagram. (**d**) Evolution of the strain diagram.

For a composite sample tested under tension, an analysis of the longitudinal strain, *ε_zz_*, field evolution on the surface was carried out. As an example, shown in Figure 4a, fields were obtained for the points marked on the loading diagram in Figure 4b. A convenient analysis tool is a strain diagram along the line, *Lz*, as shown in Figure 4c. The evolution of these diagrams as the load increased was considered (Figure 4d). The plots corresponding to the states p1–p5 are highlighted in color, the other are shown for clarity. It was found out that the primary localization was formed at a load of approximately 30% of the maximum value, *P_max_*, as evidenced by single bursts in longitudinal strain (peaks 3 and 5 in Figure 4d). As the loading progressed, bursts along the entire sample length were observed; peak 4 appeared at a load of 35% *P_max_*, then peak 6 at a load of 36% *P_max_*, and peaks 1 and 2 at a load of 38% *P_max_*. The diagrams were similar and gradually increased in the peaks, while the minimum values were changing slightly. Fracture occurred in the region with the greatest strain localization. Six strain peaks were recorded, corresponding to the stripes depicted in Figure 4a. Since these stripes were directed along the winding direction, it can be assumed that the matrix between the outer reinforcement tapes was most deformed.

**Figure 5:**
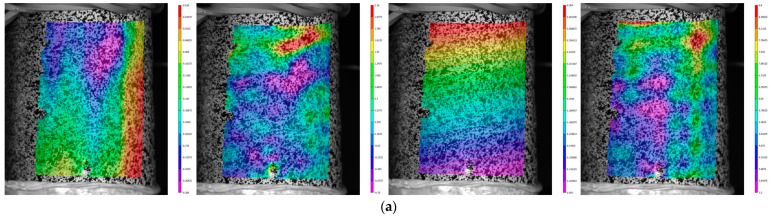

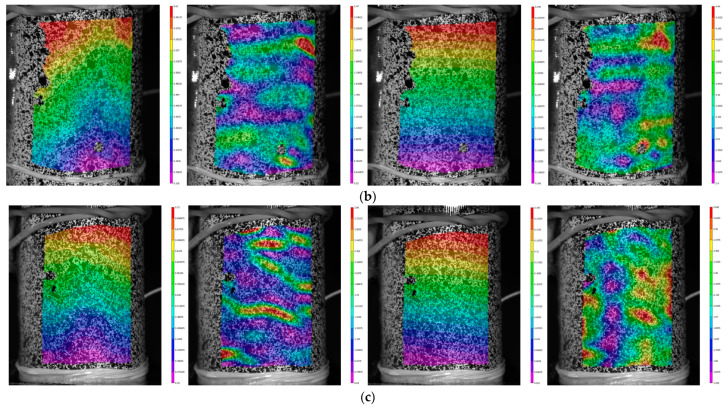
From left to right: fields of axial displacement, *u_z_*, longitudinal strain, *ε_zz_*, circumferential displacement, *u_θ_*, shear strain, *ε_θz_*, and loading modes. (**a**) Torsion. (**b**) Proportional 1. (**c**) Proportional 2.

For other loading modes, using a non-contact optical video system, the fields of axial displacement, *u_z_*, longitudinal strain, *ε_zz_*, circumferential displacement, *u_θ_*, and shear deformations, *ε_θz_*, at the maximum torque value were obtained (Figure 5). The presence of significant localization was noted, such as for the tension loading mode; however, there is no obvious formation of stripes directed along the reinforcement. The symmetry of the displacement field was maintained during loading. The necessity of the video system usage in the pipes destruction analysis was conclude since it allows us to evaluate the dangerous zones of strain localization.

**Figure 6:**
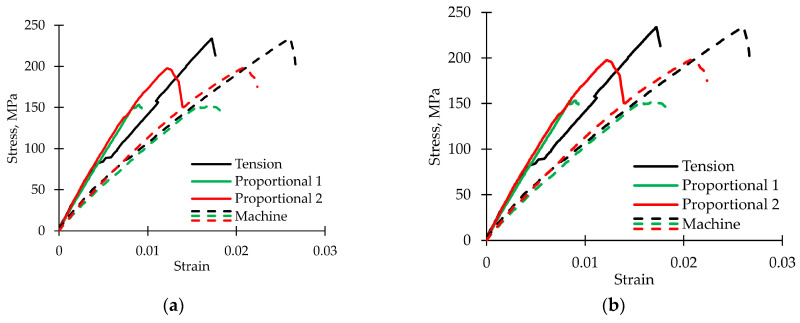
Comparison of stress–strain curves built using data from the video system and from the testing machine. (**a**) In tension. (**b**) In torsion.

Stress–strain curves were built according to the data from the video system (using the built-in module “virtual extensometer”) and from the testing machine (Figure 6). The usage of data from the machine led to an underestimation of the Young’s modulus by 1.5–1.65 times and the shear modulus by 2.1–2.25 times. However, this ratio is quite stable; therefore, it is possible to compare various loading modes of cyclic action on the change in dynamic elastic moduli.

It should be noted that in torsion tests, the material demonstrated non-linear behavior, so usage of Equation (2) in secant elastic moduli calculation seems to not be applicable [72]. However, we assumed that plastic strains were slightly influenced by the dynamic elasticity moduli, and Equation (2) still could be used in the stiffness degradation regularities. The influence of composites’ non-linear behavior on stiffness and fatigue behavior will be considered in further studies.

#### 3.1.4. Analysis of Acoustic Emission Signals

**Figure 7:**
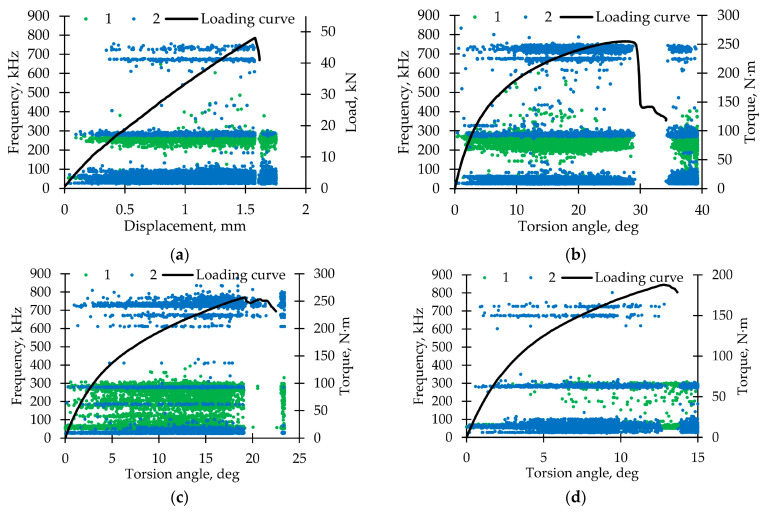
Distribution diagram of AE signal frequency parameters for loading modes. (**a**) Tension. (**b**) Torsion. (**c**) Proportional 1. (**d**) Proportional 2.

**Figure 8:**
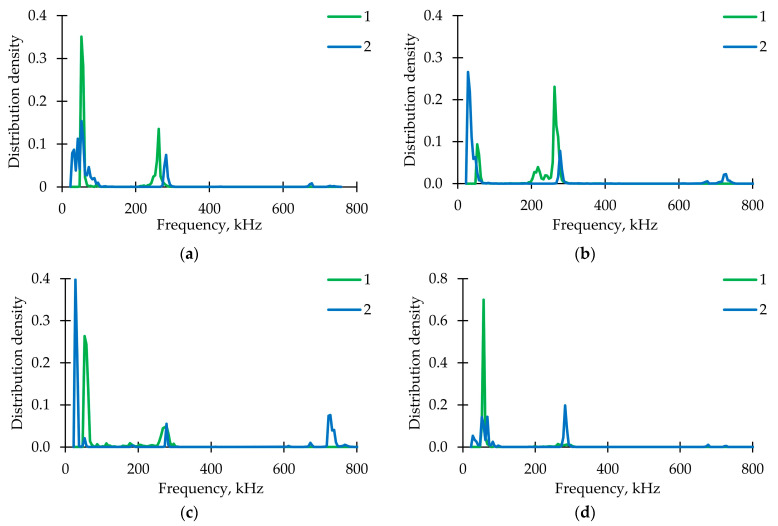
AE signal frequency distribution for loading modes. (**a**) Tension. (**b**) Torsion. (**c**) Proportional 1. (**d**) Proportional 2.

Figure 7 shows graphs of the AE signals spectral maximum frequency distribution for the fiberglass samples tested under static tension, torsion, and proportional tension with torsion. For all specimens, three frequency ranges were obtained throughout the test by sensors AE144A (No. 1, green points) and M31 (No. 2, blue points). Figure 8 represents AE signals frequency distributions. A significant part of the signals was located in the ranges up to 100 kHz (matrix cracking) and 250–300 kHz (violation of adhesion between matrix and fiber). A small number of signals were recorded in the range of 600–800 kHz (fiber breakage) by the M31 sensor. Similar frequency ranges were observed in other GFRP tests [73,74].

**Figure 9:**
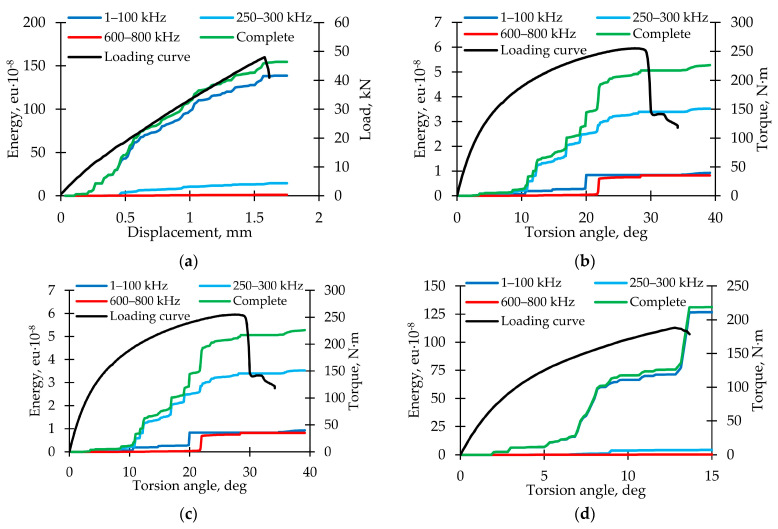
Cumulative energy for AE signals with various frequency ranges for loading modes. (**a**) Tension. (**b**) Torsion. (**c**) Proportional 1. (**d**) Proportional 2.

To assess the damage accumulation intensity, graphs of the complete cumulative energy (from sensor No. 1) and cumulative energy for frequency ranges of 1–100 kHz, 250–300 kHz, and 600–800 kHz dependences on displacement (or torsion angle) were constructed, combined with a loading diagram for all specimens (Figure 9). Various nature and rate of damage accumulations were noted for each loading mode. The complete cumulative energy value corresponded with the maximum normal stress value; an increase from ≈5 × 10^8^ eu for pure torsion up to ≈150 × 10^8^ eu for pure tension was observed. The cumulative energy curves for various frequency ranges had similar characteristics for the proportional loading modes. The greatest contribution to the AE signals complete cumulative energy in the torsion loading mode was made by the violations of adhesion (250–300 kHz range). In other loading modes, the greatest contribution was made by matrix cracking (up to 100 kHz range). In comparison, fiber breakage (600–800 kHz range) was almost absent in all of the loading modes. It can be concluded that the failure mechanism depends on the loading mode.

#### 3.1.5. Surface Defects after Static Failure

**Figure 10:**
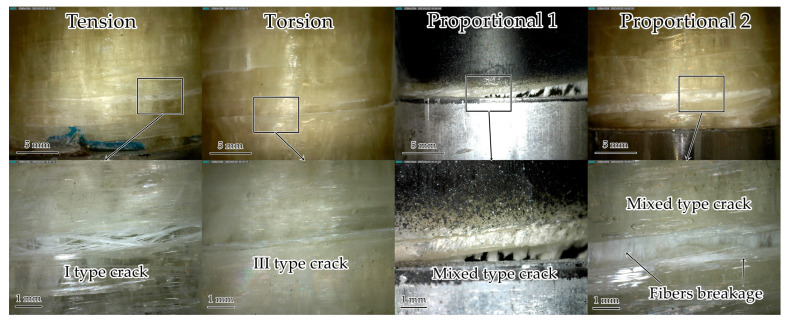
Samples after static failure.

The analysis of defects occurring on the sample surface after static failure was carried out (Figure 10). The formation of many type I cracks due to the destruction of the matrix inside the winding tape during the tension test was revealed, which corresponds with the data obtained by the acoustic emission system. During torsion, a lot of damage was obtained associated with a violation of adhesion between the matrix and the fibers, which led to the formation of type III shear cracks on the surface. A mixed-type crack was formed near the sample gripping part in the Proportional 1 loading mode due to matrix destruction and adhesion violation. Next, the destruction of the tape fibers (placed along the sample axis) occurred, which explains the peak in the 700–750 kHz frequency range of acoustic emission signals. In the Proportional 2 loading mode, mixed-type cracks and few destroyed fibers (along the winding angle and along the tube axis) were also observed. A good correspondence between acoustic emission signals analysis and optical microscopy was concluded.

### 3.2. Cyclic Loading

#### 3.2.1. Fatigue Test Results

The results of the fatigue tests are shown in Table 2. The average durability values were as follows: for uniaxial tension *N*_0_ = 40,255, for torsion *N*_0_ = 29,972, for Proportional 1 mode *N*_0_ = 4066, and for Proportional 2 loading mode *N*_0_ = 16,024. In case of equal ratios of the stress tensor components maximum values in the cycle to their values during static tests, proportional loading led to a significant decrease in the composite material durability. Besides, the Proportional 1 mode was more dangerous than Proportional 2, where the normal stress was greater. A number of cycles less than 50,000 is explained by many structural defects; maximum stress values must be reduced to increase durability. In addition, significant variations in durability were obtained; therefore, additional tests are required. These data will be used in further investigations of the reduction in the strength properties of fiberglass tubes.

#### 3.2.2. Approximation and Analysis of Fatigue Sensitivity Curves

**Figure 11:**
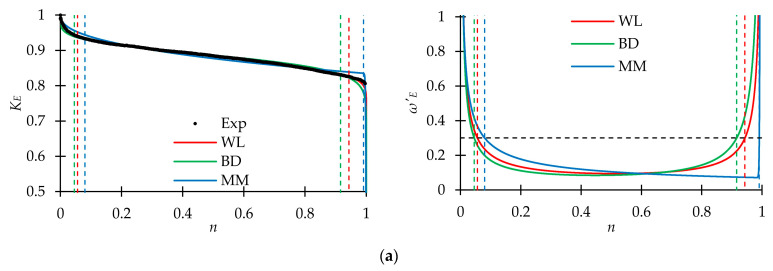

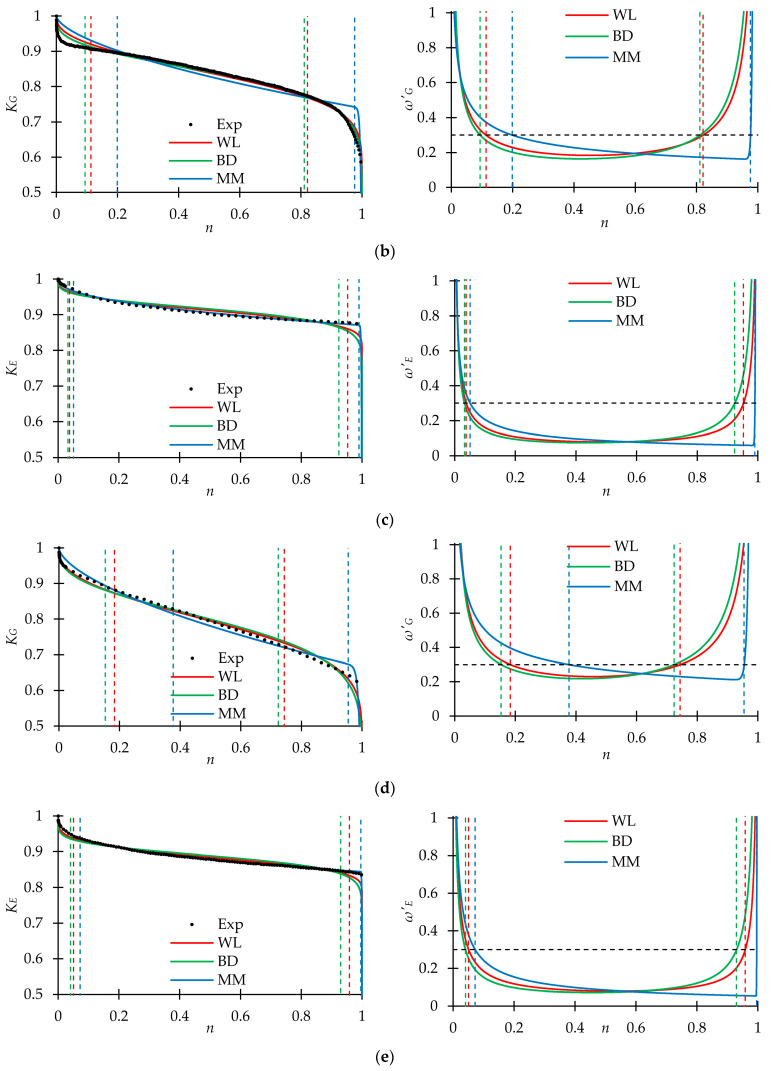

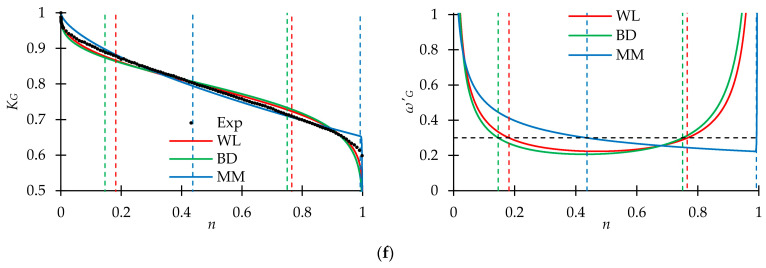
Fatigue sensitivity curves (left side) and damage value function derivatives (right side) for the dynamic elasticity modulus and loading mode. (**a**) Young’s modulus, Tension. (**b**) Shear modulus, Torsion. (**c**) Young’s modulus, Proportional 1. (**d**) Shear modulus, Proportional 1. (**e**) Young’s modulus, Proportional 2. (**f**) Shear modulus, Proportional 2.

Figure 11 represents the experimentally obtained curves using Formula (3) dependences *K_E_*(*n*) and *K_G_*(*n*) and their approximations by models based on the Weibull distribution law (WL), beta distribution (BD), and that proposed by Mao and Mahadevan (MM) (given in formula (4)). In addition, to determine the boundaries of the damage accumulation stages, diagrams of the damage value growth rate, *ω*′*_E_*(*n*) and *ω*′*_G_*(*n*), determined by Formula (5), were plotted. For all approximation variants, high values of the determination coefficient were noted: for the WL model, *R*^2^ was not lower than 0.957; for the BD model, it was not lower than 0.920; and for the MM model, it was not lower than 0.885. The high descriptive capability of approximations was concluded.

The dynamic shear modulus is sensitive to the preliminary cyclic exposure; it decreased down to 60–65% of the initial value before the onset of failure. However, this decrease was not sensitive to the stress–strain state. In addition, the sharpest drop at the initiation stage was also observed for the shear modulus in all tests (up to 90% of the initial value). On the contrary, the dynamic Young’s modulus decrease during the cyclic loading was not so sharp: down to 80% from the initial value before the failure. This dissimilarity in elastic moduli reduction can be explained, firstly, by the presence of fibers oriented along the sample axis, and secondly, by weaker adhesion between the fiber and the matrix inside the tape.

The stabilization stage predicted by the models was quite long; its length ranged from 0.6–0.7 for the shear modulus up to 0.9 for the Young’s modulus of the material. The WL and BD models gave close experimental data approximations (WL model slightly shifted the start of the stabilization stage to the right). The MM model described the experimental data well; however, it had a significant disadvantage as it strongly shifted the start of the stabilization stage to the right, but at the same time, predicted a sharp increase in the damage accumulation rate at the aggravation stage. This can lead to an over-prediction of the material elastic properties at the end of the stabilization stage. In this regard, usage of models based on the cumulative probability distribution functions is preferred.

The ratio between the maximum values of normal and shear stresses in a cycle was expressed through the parameter *η*, which lay in the range from 0° to 90° and was determined by the formula:

(8) $$\eta =\arctan\left(\frac{\sigma_{\max}}{\tau_{\max}}\right)$$

**Figure 12:**
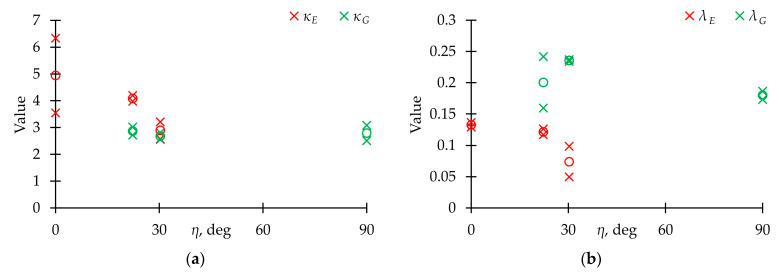

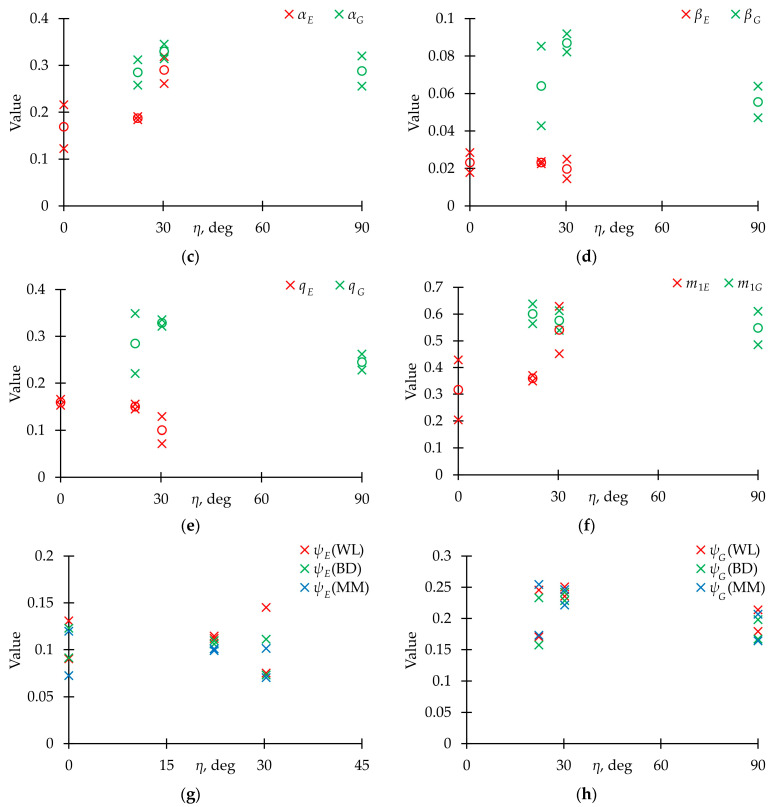
Dependence of approximation models’ parameters on *η*. (**a**–**f**) Corresponds to *κ*, *λ*, *α*, *β*, *q*, *m*_1_. (**g**) Dependence of the average rate of dynamic Young’s modulus reduction at the stabilization stage, *ψ*, on parameter *η*. (**h**) The same dependence for dynamic shear modulus. ×—experimental values, ○—average values.

The models’ parameter dependences on the *η* value are shown in Figure 12 (the parameter *m*_2_ of the MM model in all cases was a large number, and its change by 10 or more times slightly affects the shape of the fatigue sensitivity curve, so it was not shown in the figure). For the same loading mode on different samples, very close parameters values were obtained. Hence, identified patterns were reliable. The analysis shows that in some cases, the models’ parameters slightly depended on the stress state (*κ_G_*, *α_G_*, *β_E_*, *m*_1*G*_). On the other hand, several dependences were not only nonlinear, but also nonmonotonic (*λ_G_*, *β_G_*). Therefore, it is necessary to carry out additional research of the parameters dependences on the ratio between the components of the stress tensor (κ(*η*), etc.).

Figure 12 also represents the dependences of the average rates of dynamic elasticity moduli reduction at the stabilization stage, *ψ* [46], on the parameter *η*. All models gave approximately the same value of this parameter: *ψ* ≈ 0.1 for the Young’s modulus and *ψ* ≈ 0.2 for the shear modulus. It seems convenient to use the average rate of dynamic elasticity moduli reduction in composite structure design to estimate the mechanical properties during fatigue damage accumulation.

#### 3.2.3. Analysis of Acoustic Emission Signals

**Figure 13:**
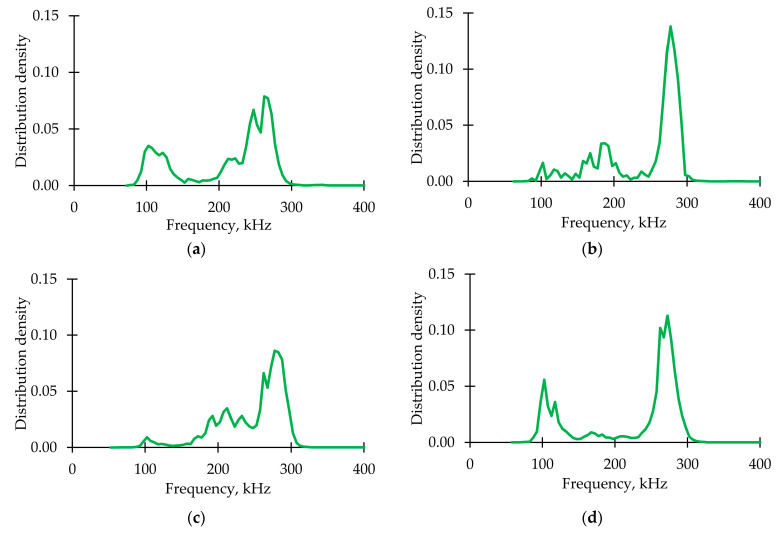
AE signals frequencies distribution for loading modes. (**a**) Tension. (**b**) Torsion. (**c**) Proportional 1. (**d**) Proportional 2.

**Figure 14:**
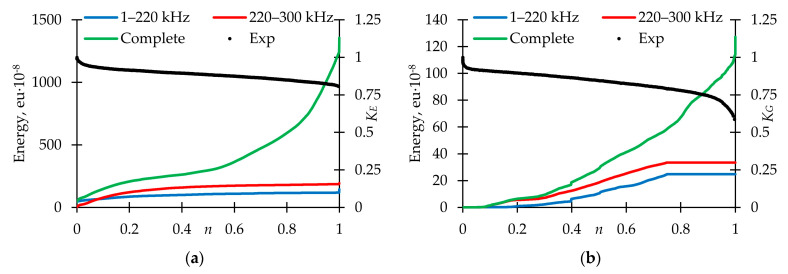

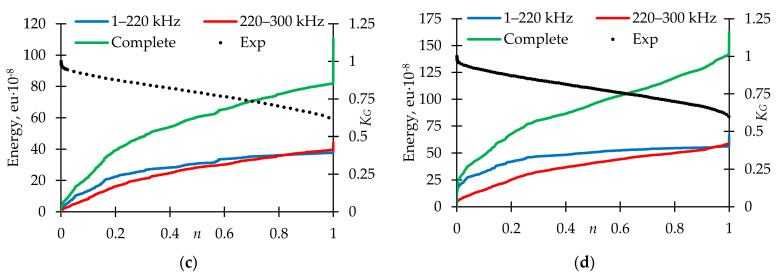
Cumulative energy for AE signals with various frequency ranges for loading modes. (**a**) Tension. (**b**) Torsion. (**c**) Proportional 1. (**d**) Proportional 2.

Figure 13 represents the signal frequency spectral maximum distribution for fiberglass specimens tested under cyclic tension, torsion, and proportional tension with torsion. Only the AE144A sensor (100–500 kHz) was used. Two typical frequency ranges were observed: up to 220 kHz (corresponds to matrix cracking) and 220–300 kHz (violations of adhesion). In comparison with static loading, frequency distributions were smooth. The largest number of signals was in the range of 220–300 kHz for each loading mode. Figure 14 shows dependences of the total cumulative energy (complete and for two described frequency ranges) on preliminary cyclic exposure. The maximum complete cumulative energy was observed for specimens tested under cyclic tension. In other cases, the maximum complete cumulative energy was similar. In addition, under proportional loading, contributions of matrix cracking and violations of adhesion to the AE signals complete cumulative energy were practically equal. The stage-by-stage nature of damage accumulation was noted. During pure tension and torsion cyclic loading, the cumulative energy growth rate was gradually increasing. On the other hand, during proportional loading, the cumulative energy growth rate was decreasing up until failure. However, transition from one stage to another was observed in all cases after preliminary cyclic exposure ≈ 0.25 N_0_. It seems appropriate to conduct further studies to establish the relationship between the damage accumulation stages and acoustic emission signals.

#### 3.2.4. Surface Defects after Fatigue Failure

**Figure 15:**
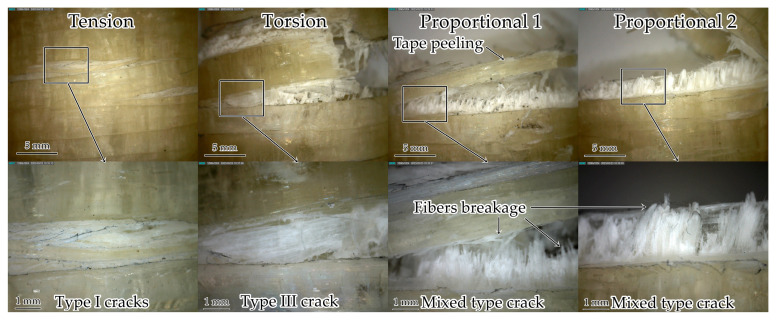
Samples after fatigue failure.

The analysis of surface defects was carried out (Figure 15). In all loading modes, the destruction occurred in the working part. The formation of multiple type I cracks during cyclic tension was revealed; it corresponded to the static failure. During cyclic torsion, a lot of adhesion violations occurred between the matrix and the reinforcing fiber (type III shear cracks). In addition, winding tapes detached one from another and broke. In the Proportional 1 loading mode, mixed type cracks were observed due to the matrix destruction and the violation of adhesion; the winding tapes also detached and broke. During the Proportional 2 cyclic loading, mixed type cracks occurred. Besides, fibers, which were placed along the sample axis, were destructed. The data on surface damage was in good agreement with the acoustic emission signals. Therefore, the acoustic emission system and optical microscopy should be used for further investigations of composites’ mechanical behavior under preliminary cyclic exposure.

## 4. Conclusions

This paper presents the results of the investigation of the mechanical behavior of thin-walled fiberglass tubes under proportional multiaxial cyclic loading. The sensitivity to the complex stress–strain state and non-linear behavior were observed. The inhomogeneous displacement and strain fields were analyzed using a non-contact optical video system. The necessity of video system usage in defining the mechanical properties of the composites was concluded. Fatigue tests were carried out under loading modes similar to those in static tests. Dependences of the fatigue sensitivity curves approximation models’ parameters on the stress–strain state were revealed. We recommend using previously proposed models [46], which are based on cumulative distribution functions, to calculate composites’ residual mechanical properties. Sharing the optical microscope and acoustic emission system established a connection between the occurring defects and acoustic response signals. The stage-by-stage nature of damage accumulation was noted. Further investigations will be aimed at defining the connection between damage accumulation stages and occurring defects.

Based on the above, we conclude the necessity and rationality of the fatigue sensitivity investigation of composites under multiaxial cyclic loading; it must be taken into account in composite structure design for accurate life prediction.

## Figures and Tables

**Table 1:** Experimental program.

Sample Number	Experiment Type	Loading	VIC-3D	AMSY-6
1	Quasi-static	Tension	+	+
2			−	−
3			−	−
4		Torsion	+	+
5			−	−
6			−	−
7		Proportional 1	+	+
8			−	−
9		Proportional 2	+	+
10			−	−
11	Cyclic	Tension	−	+
12			−	−
13		Torsion	−	+
14			−	−
15		Proportional 1	−	+
16			−	−
17		Proportional 2	−	+
18			−	−

**Table 2:** Fatigue test results.

Sample Number	Loading	*N*_0_, Cycles
11	Tension	49,681
12		30,828
13	Torsion	29,306
14		30,638
15	Proportional 1	4274
16		3858
17	Proportional 2	11,523
18		20,525

## Data Availability

Not applicable.
